# Treatment of Bloody Tumors by the Actual Cautery

**Published:** 1849-10

**Authors:** 


Art. VI.—*Treatment of Bloody Tumors by the Actual Cautery.* Translated from the Gazette des Hôpitaux, by David W. Yandell, M. D., of Louisville, Ky.

The treatment of these venous sanguine tumors, which are so frequently found in children and so rarely in adults, is far from having attained the highest degree of perfection. The two methods most commonly employed—ablation and ligature—are, as is well known, both open to very serious objections.

Ablation, the method generally in use in Germany, besides exposing to dangerous hemorrhages, is inevitably followed by a loss of substance, more or less great, according to the volume of the tumor, and leaves an indelible cicatrix. In certain localities, it is true, this inconvenience may be very insignificant; but in others again it may be sufficient to render this method entirely impracticable. An example in point is furnished by the first case which will presently be reported. In this instance, the tumor occupied the superior eyelid; in order to remove it it would have been necessary to sacrifice the greater part of the skin of this lid, which would have invited and almost certainly been followed by a considerable ectropion, that would have been not only a deformity but a very serious infirmity.

This method exposes also to phlebitis, to the development of which the peculiar tissue of which these tumors are composed, naturally predisposes.

The ligature does not expose less than ablation to this latter accident; it exposes also to hemorrhage; is slow and uncertain in its action; and, finally, it also produces the loss of a certain amount of substance.

The use of needles and setons in the treatment of these tumors is attended by all the inconveniences save the loss of substance, of either of the preceding methods and is much more uncertain in its results.

M. Lenoir of the Necker Hospital, of Paris, believes that he has found in the actual cautery a means free from these objections. Cauterization with the red hot iron possesses the property of coagulating the blood in the vessels, and in this way prevents hemorrhage. The labors of M. Bonnet, of Lyons, have proved that it exposes less than any other to phlebitis, and finally the hope was entertained that by it we might obtain the diminution and obliteration of the tumor without producing any loss of substance.

Dr. Lenoir soon had an opportunity of demonstrating the truth of these opinions. In 1844 or 1845, he brought before the Parisian Surgical Society a child affected with a bloody tumor of the size of a pullet's egg, situated in the parotid region. The tumor was traversed from one side to the other in three or four different places by a platina needle heated to a white heat. Shortly after it had diminished full three-quarters in size. Unfortunately about this time the child was lost sight of, and it was never satisfactorily ascertained whether the cure was radical or not.

Relative to this subject, Auguste Berard, who was then president of the society, and who died not very long after, a loss to science and the world, remarked that the thought of the same operation had also occurred to him, and that he had practised it with success.

Subsequently M. Lenoir, treated in like manner in a child at the breast a similar tumor situated on the forearm. In this case, the inflammation produced by the cau terization passed the desired limits; gangrene attacked a portion of the tumor, but the little patient eventually re covered without other accident.

Two other opportunities for repeating the operation having been recently afforded Dr. Lenoir, a brief transcript of them as published in the *Gazette des Hôpitaux* may not be unacceptable to the profession.

Case I. A round, well developed, healthy child, of thirteen months, has had, since it was five weeks old, a tumor seated on the right eyelid. The tumor, about the size of a chesnut, of a bluish tinge, was encircled by veins which, as the tumor, dilated when the infant cried; situated on the superior lid, towards the great angle of the eye, it embarrassed the movements of this lid and prevented its elevation.

A flat needle of platina, heated to a white heat, was repeatedly introduced into the tumor, which in consequence of the operation became inflamed, suppurated and then gradually diminished in volume. After the cauterization, simple compresses dipped in cold water were applied to the eye during twenty-four hours; these were afterwards succeeded by cataplasms. The child for some days had a slight fever and ate nothing.

The tumor was touched three times with the nitrate of silver. At the end of a month the child was cheerful and did not appear to suffer in the least. The tumor had lessened very perceptibly in size and the eye could be partially opened; otherwise healthy.

The tumor continued to decrease in volume, and in the course of another month there remained only a faint brown discoloration of the skin at the seat of the tumor, and a very slight deformity; the crease of the superior eyelid is less distinct towards the internal angle of the eye; and this opens less perfectly than that of the eye of the opposite side; this slight difference between the two eyes in a short time became still less, and manifestly tended to disappear entirely. The general health of the child was very good. It was dismissed from the hospital, cured.

The following remark concerning the above case is made, we think with much truth by the editor of the *Gazette des Hôpitaux:* Not only would no other method than the actual cautery have been followed by such satisfactory results, but it is even very doubtful whether one would have been willing to hazard the use of these methods in the region where the tumor was located.

Case II. A child, æt. 11 months, had, since the age of five months, a tumor of the size of a large nut, situated on the inferior part of the sternum. The tumor offered the character noted in the preceding; violet hue increasing in intensity during cries of the child; volume augmenting from same cause.

The needle heated to a white heat was introduced a single time; simple dressings; moderate suppuration was established. Gentle compression was now used, and the tumor gradually diminished in volume and finally completely disappeared, leaving only a slight reddish spot to mark its existence.

It will be remarked in this case, Dr. Lenoir practised cauterization only once, in order to avoid producing too high a degree of inflammation and consecutive gangrene. He will hereafter adopt this course, which in many respects is preferable to the other—repeated cauterization—since it is a very easy matter, if a single cauterization prove inefficient to repeat it a second, and if necessary at a later period, a third time. This procedure, it is true, will protract the recovery, but the loss of time is more than counterbalanced by the chances of loss of substance being thereby greatly diminished, which is of the greatest importance, especially in cases, where for example, the tumor is situated on the eyelids.

